# RUSHing back: Kinetic analysis of adaptor protein complex-1 (AP-1)–mediated retrograde traffic

**DOI:** 10.1083/jcb.202406100

**Published:** 2024-06-24

**Authors:** Mara C. Duncan

**Affiliations:** 1 Department of Cell and Developmental Biology, University of Michigan, Ann Arbor, MI, USA

## Abstract

Numerous biomedically important cargoes depend on adaptor protein complex-1 (AP-1) for their localization. However, controversy surrounds whether AP-1 mediates traffic from or to the Golgi. Robinson et al. (https://www.doi.org/10.1083/jcb.202310071) present compelling evidence that AP-1 mediates recycling to the Golgi.

## Teaser

Mara Duncan discusses work from Robinson and colleagues that monitors the entry of newly synthesized and recycled cargo into clathrin-coated vesicles associated with the adaptor protein complex-1.

The adaptor protein complex-1 (AP-1) is a conserved clathrin adaptor. 40 years ago, it and the related AP-2 complex were identified as major components of the clathrin-coated vesicles capable of assembling clathrin in vitro (reviewed in 1). AP-1 resides on intracellular organelles, whereas AP-2 is at the plasma membrane. Subsequent work firmly established that AP-2 mediates endocytosis, but the role of AP-1 has remained controversial. In this issue, Robinson et al. (2) provide insight into its role in recycling to the Golgi.

**Figure 1.**
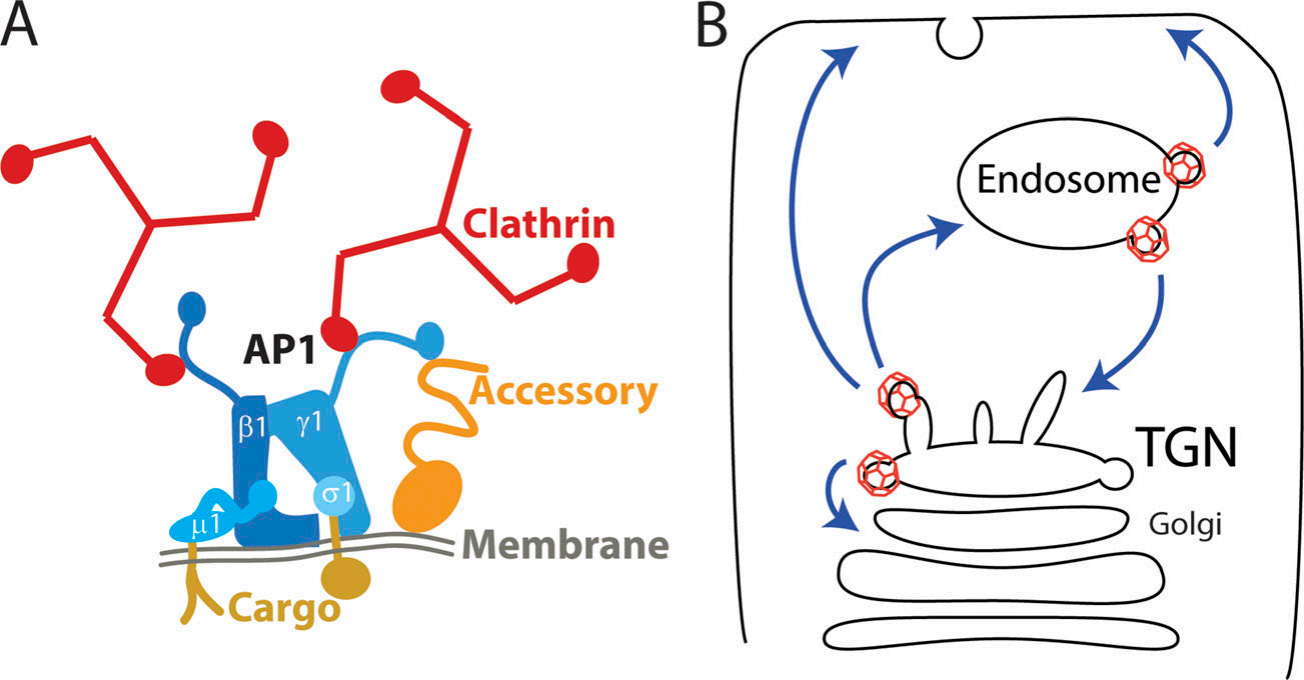
**Membrane traffic mediated by AP-1. (A)** Simplified schematic of AP-1 in linking cargo to a clathrin-coated vesicle. AP-1 is a heterotetramer composed of β, γ, µ, and σ subunits. It contains multiple cargo-binding regions and binds clathrin and other accessory coat components to promote coat assembly. **(B)** Potential AP-1–mediated trafficking routes based on inhibition studies (blue arrows). At the TGN, AP-1 has been proposed to function to mediate traffic to the plasma membrane, endosomes, and recycling to the Golgi. At the endosome, AP-1 has been proposed to mediate traffic to the plasma membrane and to the TGN.

AP-1 is a critical factor in cell physiology. It is essential in animals, and mutations that disrupt AP-1 cause genetic disorders that impact a wide range of tissues (reviewed in 3). Moreover, AP-1 is hijacked by numerous viruses (reviewed in 4). AP-1’s interactions and biochemical activities have been the subject of much study (reviewed in 5). Like all AP complexes, AP-1 is a heterotetrameric complex that selects cargo and directs it into transport vesicles or related carriers. AP-1 does this by binding to the cargo and other components of the clathrin coat, including clathrin (Fig. 1 A). AP-1 cargoes include SNARE proteins, Golgi-resident enzymes, and the mannose-6-phosphate receptors, which are receptors for newly synthesized lysosomal hydrolases. What has been less clear is where AP-1 takes this cargo.

AP-1 inhibition studies have suggested that AP-1 directs cargo from the TGN toward the endosomes, plasma membrane, or Golgi, and, at the endosome, directs cargo to the TGN or plasma membrane (Fig. 1 B). These roles are consistent with AP-1’s localization to both the TGN and the endosomes. One challenge with these inhibition studies is the nature of AP-1 cargoes, many of which cycle between numerous organelles. This causes several problems. In the absence of AP-1, other trafficking pathways can redirect the cargo, obscuring any primary effect of AP-1 on cargo localization. The cycling nature of the proteins makes their transit through any one compartment difficult to monitor. Finally, because AP-1 cargos such as the SNARES are themselves regulators of traffic, inhibition of AP-1 can indirectly impair other trafficking pathways. Therefore, observations from knock-down, knock-out, or other inhibition strategies may not reveal the actual role of AP-1.

Further complicating the understanding of AP-1 is a second class of clathrin adaptor at the TGN and endosomes, the GGA adaptors. These monomeric adaptors encode many of the same activities as AP-1, raising the potential that they are redundant with AP-1. Alternatively, work showing they interact and colocalize led to models that they function together to mediate traffic in human cells. In contrast, a picture has emerged in yeast where the two adaptor types play distinct roles: GGAs mediates traffic from the TGN to the late endosomes, and AP-1 mediates recycling within the TGN/Golgi system (6–8). However, whether this functional separation is unique to yeast’s simplified organellar system or a conserved feature of the adaptors is unclear.

In this study, Robinson et al. (2) used elegant assays to probe the role of AP-1 in exit from the Golgi and recycling from peripheral compartments. Key to this work is the use of the RUSH (Retention Using Selective Hooks) system to synchronize the release of AP-1 cargo from the ER. This powerful tool was coupled to vesicle purification and in vitro imaging to monitor the entry of newly synthesized cargo into AP-1 vesicles at different time points after cargo release from the ER. The system was further elaborated to track cargo that had reached the plasma membrane and was being recycled by AP-1. The authors utilized an extracellular nanobody that recognized the cargo to do this. These studies unambiguously show recycled cargo in the AP-1 vesicles. When coupled with prior studies that showed disruption of AP-1 causes peripheral accumulation of its cargo (9,10), a picture emerges of AP-1 mediating cargo retrieval from post-Golgi compartments, somewhat analogous to its role in yeast.

In addition, the authors used structured illumination microscopy, which provides a higher resolution than previous studies (11), to explore the relationship of AP-1 to its cargo and the GGA proteins. AP-1 is found on tubules that exit the TGN carrying at least one AP-1 cargo, a Golgi enzyme, and a lysosomal protein that is not a cargo of AP-1. However, its function on these tubules is unclear. Notably, AP-1 does not colocalize with a second cargo, the cation-dependent mannose-6-phosphate receptor, as it exits the TGN on vesicles. However, this cargo eventually enters AP-1 vesicles, presumably when it is recycled from a post-Golgi compartment. These findings argue against the role of AP-1 in forming vesicles at the TGN carrying these two cargoes toward the plasma membrane or endosomes.

Significantly, when exploring the relationship with GGAs, the authors find that although AP-1 and Gga2 concentrate in the same region of the cell, they rarely colocalize. Moreover, Gga2 is absent from purified AP-1 vesicles. These data argue that the AP-1 and GGAs do not cooperate to form the clathrin-coated vesicles isolated in this study.

This work provides clear evidence for the role of AP-1 in recycling and argues against cooperation between AP-1 and GGAs in exit from the TGN, at least in the HELA cells used in this study. Importantly, the tools used will be highly useful for studies on other types of vesicles in the cell. However, many questions remain unanswered. A substantial pool of AP-1 localizes to the TGN; what is it doing if it is not forming vesicles? What is the role of AP-1 on tubules emerging from the TGN? Potential roles include recycling cargo back to the Golgi or in the forward traffic of cargo that this study did not monitor. Indeed, the study looked only at the cargo of vesicles that retained AP-1 throughout a multi-step purification; therefore, the function of AP-1 on tubules or on vesicles that uncoat rapidly was missing. In addition, there are multiple AP-1 complexes in human cells. These complexes derive from alternate genes encoding three of the four AP-1 subunits. Notably, this study only investigated the AP-1 complexes containing the γ subunit encoded by AP1G1. Do AP-1 complexes defined by the other γ subunit share the same function in recycling? The tools described here will be ideal for exploring the roles of AP-1 complexes with different compositions or in other cell types, where differential expression of AP-1 binding partners may reshape its roles. Therefore, this study reveals new insight into AP-1 and lays the foundation for many future discoveries.
